# Efficacy of the external oblique intercostal plane block for postoperative analgesia after laparoscopic radical nephrectomy

**DOI:** 10.55730/1300-0144.6146

**Published:** 2025-11-13

**Authors:** Gizem AVCI, Yusuf ÖZGÜNER, Damla USALAN, Cem Koray ÇATAROĞLU, Habip YILMAZ, Savaş ALTINSOY, Jülide ERGİL

**Affiliations:** 1 Department of Anesthesiology and Reanimation, Ankara Etlik City Hospital, Ankara, Turkiye; 2 Department of Anesthesia and Reanimation, Sultan Abdulhamid Training and Research Hospital, Health Sciences University, İstanbul, Turkiye

**Keywords:** External oblique intercostal block, laparoscopic radical nephrectomy, postoperative pain

## Abstract

**Background/aim:**

The external oblique intercostal plane block (EOIB) is a regional anesthesia technique that may be utilized for the management of postoperative pain following laparoscopic radical nephrectomy. This study aims to evaluate the efficacy of EOIB after laparoscopic radical nephrectomy by comparing 24 h tramadol consumption between groups.

**Materials and methods:**

Initially, 66 patients were enrolled in the study, and after exclusions, 60 were analyzed and divided into two groups: the EOIB group and the control group. Postoperative tramadol consumption, pain scores at rest and during movement, and dermatomal coverage of the block were recorded.

**Results:**

The EOIB group (181.33 ± 57.038 mg) showed significantly lower tramadol consumption compared to the control group (283.33 ± 49.013 mg; p < 0.05). In the EOIB group, numerical rating scale scores at 0, 2, 4, and 6 h were significantly lower both at rest and during movement (p < 0.05).

**Conclusion:**

EOIB resulted in lower tramadol consumption compared to the control group. These findings indicate that EOIB is an effective regional anesthesia technique that can enhance postoperative analgesia.

## 1. Introduction

Laparoscopic nephrectomy is a widely performed urological procedure associated with faster recovery, fewer complications, and shorter hospital stays compared to open surgery [1]. However, postoperative pain—which involves both somatic and visceral components—remains a significant clinical challenge. This can be attributed to several factors. These factors include phrenic nerve neuropraxia caused by excessive stretching, residual intraperitoneal gas, the properties of the insufflated gas (type and temperature), carbon dioxide–induced acidosis, the extent of surgical incision, and the use of postoperative drainage systems [2].

Multimodal analgesia protocols based on the enhanced recovery after surgery (ERAS) society guidelines aim to reduce postoperative pain and improve patient satisfaction, demonstrating a significant association with reduced opioid consumption within the first 24 h, earlier mobilization, and a lower risk of opioid-related complications [3]. Traditional pain management strategies, including epidural analgesia, can be effective but are associated with potential complications such as hypotension, infection, and limitations in anticoagulated patients [4,5]. Consequently, increasing interest has been directed toward alternative, less invasive regional anesthesia techniques such as the transversus abdominis plane (TAP) block, quadratus lumborum block (QLB), erector spinae plane (ESP) block, and, more recently, the external oblique intercostal plane block (EOIB) [6].

EOIB was first described by Hamilton et al. [7], who demonstrated that the block affects both the anterior and lateral branches of the T7–T10 intercostal nerves and produces a dermatomal sensory block extending from T6 to T10 along the anterior axillary line and from T6 to T9 at the midline [8].

The present study evaluates the efficacy of EOIB in managing postoperative pain following laparoscopic radical nephrectomy.

## 2. Materials and methods

### 2.1. Ethical statement

1This prospective, randomized study was approved by the Ethics Committee of Ankara Etlik City Hospital (approval no: 0066) and conducted between June and October 2024. The study was registered in the ClinicalTrials.gov registry under the identifier NCT06639035.

### 2.2. Study design

A total of 66 patients who underwent nephrectomy were included in the study. Patients younger than 18 or older than 80 years, those with severe comorbidities, a history of bleeding diathesis, infections at the block site, emergency surgery, or chronic opioid use were excluded from the study.

Allocation concealment was maintained using sequentially numbered, sealed, opaque envelopes prepared by an independent researcher. The EOIB was performed by two anesthesiologists, each with more than 5 years of experience.

### 2.3. Block procedures

**Figure 1:**
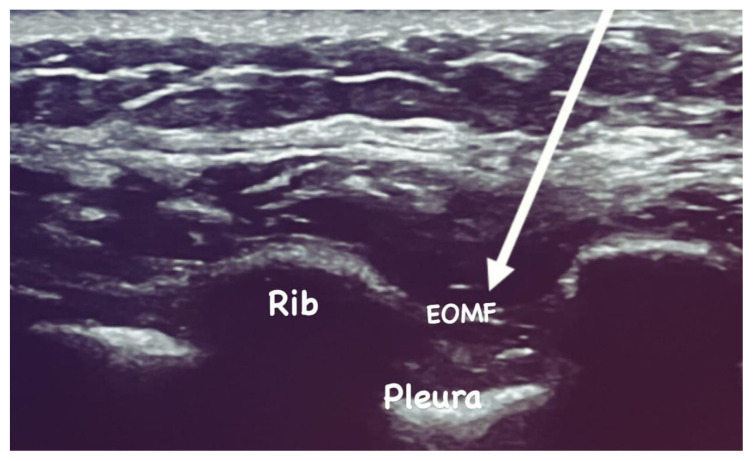
Sonoanatomy illustrating the EOIB.

The external oblique intercostal plane block was performed after induction of anesthesia using a linear ultrasound transducer (LOGIQ e; GE HealthCare, Buckinghamshire, United Kingdom) and a linear radiofrequency probe (6–13 MHz) positioned in the sagittal plane at the level of the sixth rib, 1–2 cm medial to the anterior axillary line, with the patient in the supine position. The ultrasound image allowed visualization of the ribs, lungs, pleura, intercostal muscles, external oblique muscle, and subcutaneous tissue. An inplane approach with a 22G, 80 mm block needle (Stimuplex; B. Braun Medical, Melsungen, Germany) was used. Hydrodissection was initiated by administering a test dose of saline to confirm correct separation of the muscle plane. A total of 20 mL of local anesthetic solution—comprising 10 mL of 0.5% bupivacaine (maximum 2.5 mg/kg), 5 mL of 2% lidocaine (maximum 2 mg/kg), and 5 mL of isotonic saline—was injected bilaterally, 20 mL per side (Figure 1). The operation was performed using a conventional laparoscopic technique with four approaches. Because trocar incisions, extraction sites, and pneumoperitoneum created in the flank position during laparoscopic nephrectomy may cause pain on the contralateral side, the block was therefore performed bilaterally.

Before discontinuation of the remifentanil infusion, 100 mg of tramadol, 1 g of paracetamol, and 1 mg of granisetron were administered. Tracheal extubation was performed after administration of sugammadex and confirmation of complete reversal of residual neuromuscular blockade.

The extent of dermatomal sensory blockade in patients who received the block was assessed by the attending anesthesiologist in the post-anesthesia care unit (PACU) using the pinprick method. For postoperative pain management, all patients received a patient-controlled analgesia (PCA) device containing tramadol (5 mg/mL), programmed with a 4 mL bolus dose, a 20 min lockout interval, no basal infusion, and a maximum hourly tramadol dose of 60 mg, delivered via a CADD-Legacy PCA pump (Smiths Medical, Minneapolis, MN, USA). Pain at rest and during movement was assessed by the anesthesiologist in the PACU using the numeric pain scale (NRS). The primary outcome of the study, total tramadol consumption, was determined from data recorded by the PCA device. Patients’ vital signs and NRS pain scores at rest and during movement were recorded at 0, 2, 4, 6, 12, and 24 h postoperatively. Patients routinely received 1 g of paracetamol at 8 h intervals. Patients with an NRS score greater than 4 received 50 mg of intravenous dexketoprofen. If the NRS score remained above 4 after 30 min, an additional 50 mg of intravenous tramadol was administered alongside ongoing PCA therapy. All patients were transferred to the ward without complications, and total 24 h tramadol consumption via PCA was subsequently analyzed.

### 2.4. Statistical analysis

The sample size was calculated using G*Power (Heinrich Heine University Düsseldorf, Düsseldorf, Germany) based on the preliminary data on average tramadol consumption. The mean tramadol consumption was 212 ± 48.16 mg in the EOIB group and 260 ± 58.31 mg in the control group (α = 0.05, power = 95%, effect size = 0.89). Based on this calculation, a minimum of 56 participants was required; therefore, 66 patients were enrolled to account for potential dropouts.

Statistical analyses were conducted using SPSS software (version 21.0; IBM Corp., Armonk, NY, USA). Numerical data were expressed as mean ± standard deviation or as median (Q1: first quartile–Q3: third quartile). The Kolmogorov–Smirnov test was used to determine data normality. The chi-square test (for categorical variables), independent t-test (for normally distributed continuous variables), and Mann–Whitney U test (for nonnormally distributed continuous variables) were used in this study. A p-value < 0.05 was considered statistically significant.

## 3. Results

**Figure 2:**
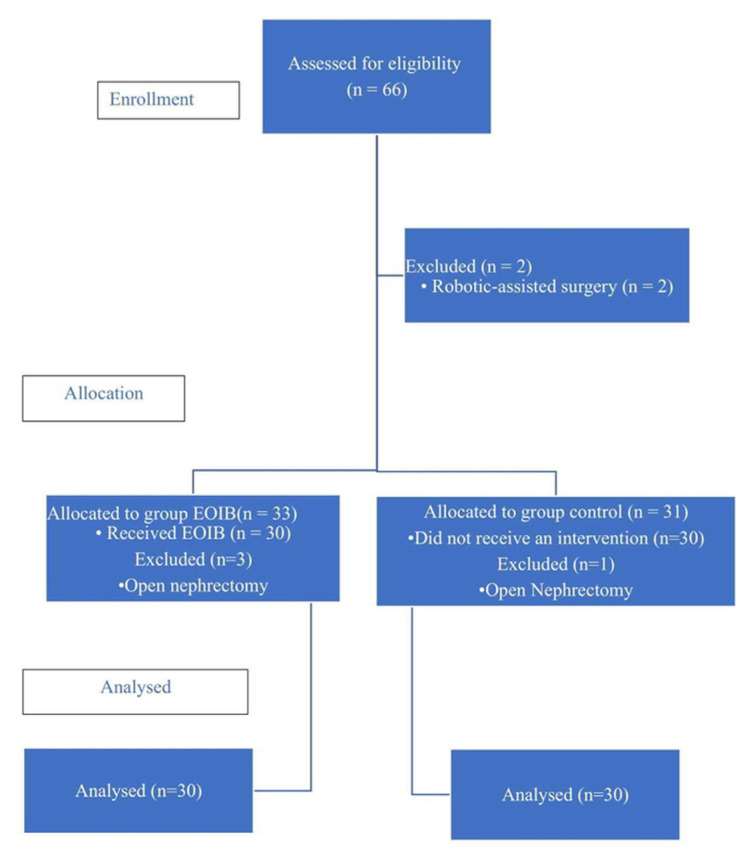
CONSORT flow diagram showing patient enrollment, allocation, follow-up, and analysis.

A total of 66 patients were initially enrolled in the study. However, two patients were excluded because they were scheduled for robotic surgery, and four patients were converted from laparoscopy to open surgery for intraoperative reasons. Consequently, 60 patients were included in the final analysis (Figure 2).

Patient characteristics—including age, American Society of Anesthesiologists (ASA) classification, smoking status, sex, body mass index (BMI), and comorbidities—as well as surgical characteristics such as operative duration were comparable between the two groups (Table 1).

The EOIB group demonstrated significantly lower tramadol consumption (181.33 ± 57.038 mg) than the control group (283.33 ± 49.013 mg; p < 0.001). Additional postoperative analgesic requirement was observed in three patients in the EIOB group and nine patients in the control group (p = 0.053).

NRS scores at rest were lower in the EOIB group than in the control group at 0, 2, 4, and 6 h postoperatively. Similarly, NRS scores during movement were significantly lower in the EOIB group up to 12 h postoperatively, with comparable values observed between groups at 12 and 24 h (p = 0.709 and p = 0.690, respectively; Table 2).

At the end of 24 h, no block-related or systemic complications were observed. Specifically, there were no cases of hematoma, pneumothorax, or systemic local anesthetic toxicity. Additionally, no opioid-induced respiratory depression occurred. The incidence of postoperative nausea and vomiting was similar between the two groups.

## 4. Discussion

Our findings demonstrate that EOIB provides effective analgesia during the early postoperative period following laparoscopic radical nephrectomy.

The significant reduction in tramadol consumption and lower NRS scores during the first 6 h suggest that EOIB provides a meaningful clinical benefit in the acute phase of postoperative pain management.

These results are consistent with previous findings in abdominal surgeries. Elsharkawy et al. demonstrated that EOIB provides sensory blockade spanning the T6–T10 dermatomes, which correspond to the typical pain distribution after nephrectomy [8]. Bansal et al. validated the analgesic benefit of EOIB in open renal surgery and reported a reduction in opioid consumption [9]. Somatic sensation of the upper abdominal wall primarily arises from the lateral and anterior cutaneous branches of the lower intercostal nerves (T6–T10); therefore, targeting these nerves can provide effective postoperative analgesia [10]. In our study, similar dermatomal distribution was evaluated using the pinprick method, and opioid consumption was found to be lower in the EOIB group.

Coşarcan and colleagues found that the block was effective in two cases of laparoscopic liver surgery and in one case of bariatric surgery [11]. Owing to its ease of use and clinical benefits, Kavakli and colleagues employed morphine PCA for postoperative analgesia in bariatric surgery. They reported that opioid consumption was lower in the EOIB group [12]. In addition, EOIB was compared with the modified thoracoabdominal nerve block through perichondrial approach in laparoscopic sleeve gastrectomy, and no significant difference was observed in 24 h morphine consumption [13]. EOIB was administered to a patient who experienced severe pain (VAS 7/10) on the second postoperative day after distal pancreatectomy. Although pain management with a thoracic epidural catheter had been unsuccessful, bilateral EOIB catheter placement resulted in a marked reduction in the patient’s pain score [14]. Given its distance from the surgical field, EOIB can be considered a viable and safe regional anesthesia technique for postoperative pain management following nephrectomy. EOIB has also been successfully applied in a case of chronic postoperative neuropathic pain, providing effective analgesia. Its simplicity of application and classification as a superficial fascial plane block make EOIB a safe option for patients receiving anticoagulant therapy [15].

Several studies in the literature have investigated postoperative pain management after nephrectomy, including the use of TAP block [16,17], QLB [18–20], and ESP block [21,22]. In contrast to TAP block and QLB, EOIB provides more consistent coverage of the lateral abdominal wall, which is particularly important due to the port placements in laparoscopic nephrectomy. The subcostal TAP block has been described as more suitable for supraumbilical surgery [23]. The analgesic effect of the subcostal TAP block extends to the upper abdominal dermatomes (T6–T9). However, this approach primarily anesthetizes the midline abdominal region, whereas the lateral regions are not adequately blocked, thereby limiting its capacity to provide full coverage of surgical areas above the umbilicus [24]. Because the subcostal TAP block cannot effectively anesthetize the lateral upper abdominal regions, its use is limited in nephrectomy, cholecystectomy, and liver surgery [25]. Unlike ESP and QLB, EOIB offers a potential advantage because it is easy to perform and does not require specific patient positioning. In a study investigating ESP blockade in patients undergoing laparoscopic nephrectomy, 24 h opioid consumption was reported as 114 mg in the block group and 212 mg in the control group [26]. Opioid medications are among the most commonly used agents for postoperative pain management through modulation of both central and peripheral nociceptive pathways [27]. When intravenous analgesia is required in the postoperative period, patient-controlled analgesia (PCA) is one of the most commonly preferred methods of opioid delivery. Interestingly, although opioid consumption was slightly higher in the PCA group, no increase in opioid-related adverse effects was observed [28]. Tramadol was chosen for postoperative pain management due to its additional efficacy in treating neuropathic pain [29]. In this study, tramadol was used for postoperative analgesia, and multimodal analgesia protocols were implemented, including the administration of paracetamol and nonsteroidal antiinflammatory drugs (NSAIDs) when additional analgesia was required.

Interestingly, opioid consumption in our EOIB group, although significantly lower than in controls, remained relatively high (mean: 181.33 mg of tramadol). Blocks such as ESP and QLB provide both somatic and visceral analgesia, whereas EOIB primarily targets somatic pain. In laparoscopic surgeries, however, postoperative pain originates from both somatic and visceral components. The absence of a visceral analgesic component in EOIB may explain the relatively higher analgesic consumption observed in our study.

A recent study comparing the ESP block and EOIB demonstrated that the analgesic efficacy of EOIB was noninferior to that of the ESP block in managing postthoracotomy pain [30]. Consistent with recent evidence, postoperative pain scores were reported to be lower with QLB than with EOIB in patients undergoing laparoscopic cholecystectomy. However, EOIB may still serve as a feasible and effective alternative, particularly in patients for whom QLB is contraindicated or technically challenging [31]. This suggests that although EOIB contributes substantially to pain relief, it should be viewed as a component of multimodal analgesia rather than a standalone solution. Its superficial anatomical location and ultrasound-guided application minimize the risk of vascular puncture or inadvertent organ injury.

This study has several limitations. The absence of a sham block in the control group may have introduced performance bias, while the relatively short observation period limits the ability to evaluate longer-term analgesic effects or the potential development of chronic pain. Individual variability in pain perception, which may influence the need for analgesics after surgery, was not taken into account. In addition, the procedure was performed by a limited number of anesthesiologists, which may have introduced operator-dependent variability.

In conclusion, this study demonstrates that EOIB reduces postoperative tramadol consumption in patients undergoing laparoscopic radical nephrectomy compared with conventional analgesic methods. In addition to reduced opioid requirements, the EOIB group also exhibited lower pain scores. These findings indicate that EOIB is an effective regional anesthesia technique capable of enhancing postoperative analgesia.

## Figures and Tables

**Table 1:** Demographic and clinical characteristics of the study participants.

	EOIB group (n=30)	Control group (n=30)	p-value

Age	59.10±10.307	58.60±11.628	0.861

ASA I	9	7	0.449
II	19	17	
III	2	5	
IV	0	1	

Sex, female/male	13/17	15/15	0.605

BMI, kg/m^2^	28.5±5.686	27.86±6.719	0.876

Duration of surgery, min	213.333±69.2737	238±64.0797	0.158

Duration of anesthesia, min	251.833±74.0746	268.233±67.9998	0.375

Smoking	10	10	1

Coronary artery disease	6	8	0.542

Hypertension	12	12	1

Diabetes mellitus	6	6	1

Values are presented as mean ± SD and numbers. n: number, BMI: body mass index; ASA: American Society of Anesthesiologists.

**Table 2:** Postoperative tramadol consumption and pain scores at rest and during movement.

	Group EOIB n=30	Group control n=30	p-value

**Tramadol consumption**	181.33 ± 57.038 mg	283.33 ± 49.013 mg	**<0.001**

**NRS scores at rest**			
T0	3 (3–4)	4 (4–5.25)	**0.002**
T2	2 (2–3)	3 (3–4.25)	**<0.001**
T4	3 (2–3)	3 (3–4)	**0.002**
T6	2 (2–2)	3 (2–4)	**<0.001**
T12	2 (1.75–3)	2 (2–3)	0.382
T24	2 (2–2)	2 (2–3)	0.905

**NRS scores during movement**			
T0	4.5 (4–5)	5 (5–6)	**<0.001**
T2	3 (2.75–3)	5 (4–5)	**<0.001**
T4	3 (2–4)	4 (4–5)	**<0.001**
T6	2 (2–3)	4 (3–5)	**<0.001**
T12	3 (2.75–4)	3 (3–4)	0.709
T24	3 (3–3.25)	3(2–4)	0.690

NRS: numerical rating scale. Values are given as median (Q1: first quartile–Q3: third quartile) and numbers. p < 0.05 was considered statistically significant. Significant values are indicated in bold.
